# Purification, Characterization, and Application for Preparation of Antioxidant Peptides of Extracellular Protease from *Pseudoalteromonas* sp. H2

**DOI:** 10.3390/molecules24183373

**Published:** 2019-09-16

**Authors:** Dan Liu, Jiafeng Huang, Cuiling Wu, Congling Liu, Ran Huang, Weng Wang, Tingting Yin, Xiaotao Yan, Hailun He, Leilei Chen

**Affiliations:** 1 School of Life Sciences, Central South University, Changsha 410013, China; 2 Department of Biochemistry and Molecular Biology, Guangxi Medical University, Nanning 530021, China; 3 Department of Biochemistry, Changzhi Medical College, Changzhi 046000, China; 4 Beijing Advanced Innovation Center for Food Nutrition and Human Health, Beijing Technology and Business University (BTBU), Beijing 100048, China; 5 Institute of Agro-Food Science and Technology, Shandong Academy of Agricultural Sciences, Jinan 250100, China

**Keywords:** *Pseudoalteromonas*, protease, hydrolysis, collagen, antioxidant peptides

## Abstract

The study reported on the isolation of a metalloprotease named EH2 from *Pseudoalteromonas* sp. H2. EH2 maintained more than 80% activity over a wide pH range of 5–10, and the stability was also nearly independent of pH. Over 65% activity was detected at a wide temperature range of 20–70 °C. The high stability of the protease in the presence of different surfactants and oxidizing agents was also observed. Moreover, we also investigated the antioxidant activities of the hydrolysates generated from porcine and salmon skin collagen by EH2. The results showed that salmon skin collagen hydrolysates demonstrated higher DPPH (1,1-diphenyl-2-picrylhydrazyl) (42.88% ± 1.85) and hydroxyl radical (61.83% ± 3.05) scavenging activity than porcine skin collagen. For oxygen radical absorbance capacity, the hydrolysates from porcine skin collagen had higher efficiency (7.72 ± 0.13 μmol·TE/μmol). Even 1 nM mixed peptides could effectively reduce the levels of intracellular reactive oxygen species. The two types of substrates exerted the best antioxidant activity when hydrolyzed for 3 h. The hydrolysis time and type of substrate exerted important effects on the antioxidant properties of hydrolysates. The hydrolyzed peptides from meat collagens by proteases have good antioxidant activity, which may have implications for the potential application of marine proteases in the biocatalysis industry.

## 1. Introduction

Proteases are enzymes that hydrolyze peptide bonds of proteins [1]. They are the most important and commonly used enzymes in industry, contributing approximately 60% of the total market of industrial enzymes [2], which includes detergents [2], anti-biofilm agents [3], and feather processing, feed processing, wastewater treatment, and food processing enzymes [4]. Microorganisms can produce extracellular proteases, and culturing and genetic manipulation of high-producing protease strains to generate novel enzymes is relatively easy. Therefore, using microorganisms to produce proteases has many advantages, such as a wider range of sources, simple operation, high performance, cost-effectiveness, and industrial production. Marine is a unique habitat with lower temperature and higher salinity. At present, marine microorganisms have gained considerable attention as an excellent source of enzymes.

*Pseudoalteromonas* can synthesize potentially valuable biologically active compounds. *Pseudoalteromonas* is the predominant protease-producing group of the cultured marine bacteria [5] and show high protease-producing ability in the marine environment [6]. Wu et al. purified an alkaline protease from the extracellular proteases of *Pseudoalteromonas*, which showed valuable feathers as an additive in laundry detergent and nontoxic anti-biofilm agents [7]. Chen et al. demonstrated that serine protease secreted by *Pseudoalteromonas* can effectively degrade collagen to prepare antioxidant peptides [8]. However, the application of metalloproteinases secreted by *Pseudoalteromonas* is uncommon, especially in the preparation of antioxidant peptides. Some metallocollagenolytic proteases have alkali- and salt-resistance. They also have multiple cleavage sites on soluble collagen. In addition, most of the metalloproteinases derived from *Pseudoalteromonas* contain PPC (pre-peptidase C-terminal) domains. Huang et al. have shown that the PPC domain could play a synergistic role in binding and swelling and assist with the hydrolysis of collagen [9]. In sum, metalloproteinases can efficiently hydrolyze soluble collagen.

Animal skins are a rich source of collagen with a variety of bioactivities. However, they are often discarded as byproducts of the meat processing industries [10] and cause serious waste of protein and environmental problems. Many studies have reported that enzymatic hydrolysis of collagen is an effective way to obtain antioxidant peptides [11,12]. Most of the proteases used in these studies are commercially available, such as trypsin, pepsin, α-chymotrypsin, papain, Protamex, and neutral protease. However, there are few reports on noncommercially resistant organic solvent alkaline proteases for antioxidant peptide preparation. Therefore, it is of great significance to search and prepare proteases that are stable and active under multiple extreme conditions, such as alkaline pH, high salt concentration, wide temperature ranges, and organic solvent.

In this study, we purified the metalloproteinase produced by *Pseudoalteromonas* sp. H2 and characterized its enzymatic properties. In addition, the potential application of the protease to hydrolyze low-value protein resources to prepare antioxidant peptides was also explored. This study may have implications for the potential application of marine proteases in the biocatalysis industry. 

## 2. Results and Discussion

### 2.1. Screening of Strain with High Protease-Producing Ability

**Figure 1:**
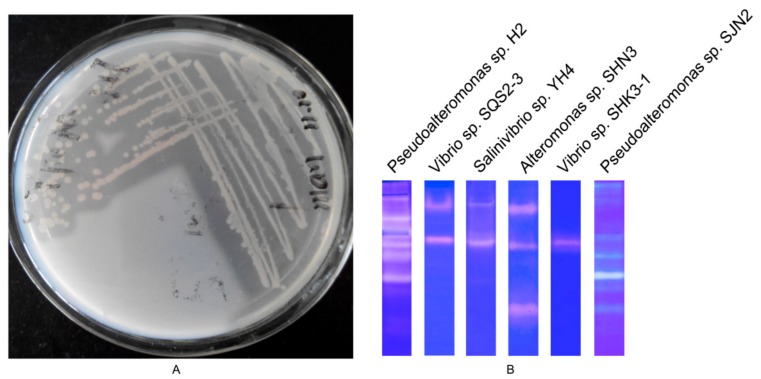
Screening of *Pseudoalteromonas* sp. H2. (**A**). Formation of clear zones on a casein plate of strain H2. (**B**). Comparison of the caseinolytic profile from *Pseudoalteromonas* sp. H2, *Vibrio* sp. SQS2-3, *Salinivibrio* sp. YH4, *Alteromonas* sp. SHN3, *Vibrio* sp. SHK3-1, and *Pseudoalteromonas* sp. SJN2.

Proteolytic activity was evaluated by the ability to form clear zones on casein plates. The strain H2 exhibited high protease-producing ability (Figure 1A). Compared with other marine strains, the caseinolytic profile of *Pseudoalteromonas* sp. H2 has some advantages in terms of the number and brightness of the strips (Figure 1B). On the basis of the superior matching with *Pseudoalteromonas* sp. CF6-1 (FJ169996.1) by the 16S rDNA (ribosomal deoxyribonucleic acid) sequences (99% identity), this strain was designated *Pseudoalteromonas* sp. H2. The 16S rDNA sequences of this strain have been submitted to GenBank (No. KX458245). *Pseudoalteromonas* can synthesize potentially valuable biologically active compounds [3] and show high protease-producing ability in the marine environment [5]. Wu et al. reported that *Pseudoalteromonas* from seawater exhibited activity and stability when the temperature and pH were varied or in the presence of surfactants and chelating agents [7]. Therefore, separating and preparing the extracellular proteases with multiple tolerances from *Pseudoalteromonas* sp. H2 would be useful for future applications.

### 2.2. Protease Production and Purification

**Figure 2:**
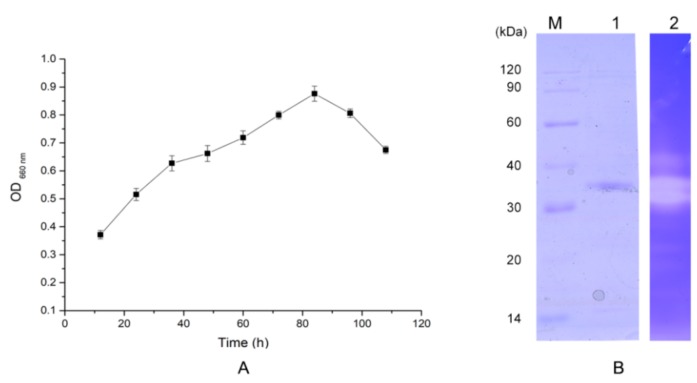
(**A**). Time-related changes in the relative activities of extracellular protease produced by *Pseudoalteromonas* sp. H2. by the Folin phenol method. (**B**). SDS–PAGE and zymography analyses of EH2. Line marked 1, SDS-PAGE; line marked 2, casein immersing zymography. Values are expressed as the mean ± SD (*n* = 3).

The time-related changes in the proteases produced by H2 were analyzed by the Folin phenol method. In Figure 2A, the strain started to secrete proteases in the first 12 h, and protease production reached a maximum after culture for 84 h. After 4 days, the enzyme activity tended to decrease. Therefore, when purifying the protease, the culture supernatant of *Pseudoalteromonas* sp. H2 was collected after 84 h of incubation and purified sequentially with ammonium sulfate precipitation, anion exchange chromatography on a HiTrap^TM^ DEAE (diethyl-aminoethyl) column, and gel filtration on a Superdex 75. The purification results are shown in Table 1. With a yield of 27%, the purified protease had a specific caseinolytic activity of 475.2 U/mg. A single band with an approximate molecular mass of 35 kDa was obtained by SDS-PAGE (Figure 2B). Similar molecular masses represented other previously characterized metalloproteases, but the specific activity of the purified EH2 was higher [13]. Zymography also gave a high degree of consistency with SDS-PAGE analysis. Further confirmation of the biological activity and purity of this protease, EH2, is shown in Figure 2B.

### 2.3. Characterization of EH2

#### 2.3.1. Effect of Temperature and pH on Protease Activity and Stability

**Figure 3:**
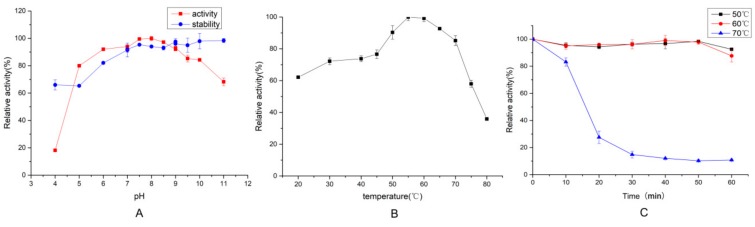
Effects of pH (**A**), temperature (**B**) on EH2 activity, and thermal stability of EH2 at 50 °C, 60 °C and 70 °C (**C**). Values are expressed as the mean ± SD (*n* = 3).

EH2 was active over a wide pH range. As shown in Figure 3A, more than 80% of the activity was detected in a range of pH 5–11, and the maximum activity occurred at pH 7.5–8. This result was comparable to other *Pseudoalteromonas* strains [13,14]. Furthermore, the stability of the protease was also nearly independent of pH (Figure 3A), and more than 90% of the relative activity was exhibited after 24 h at pH 6–11. EH2 displays multifaceted potential at different pH values, making this enzyme interesting for industrial applications.

In addition, optimal activity of the protease was observed at 60 °C (Figure 3B), with more than 85% relative activity at 70 °C. Some proteases from *Pseudoalteromonas* also displayed optimum activity at 30 °C [13,14] or 55 °C. This difference was possibly related to the genetic and environmental adaptation of strains. A rise in temperature could enhance the velocity of enzymatic reactions. However, the higher the temperature, the more easily the enzyme was inactivated. EH2 displayed good thermal stability at 50 °C and 60 °C (Figure 3C). The protease maintained more than 65% of its original activity over a wide temperature range of 20–70 °C. EH2 exhibited alkaline resistance and temperature stability.

#### 2.3.2. Effect of Inhibitors and Metal Ions on Protease Activity

**Figure 4:**
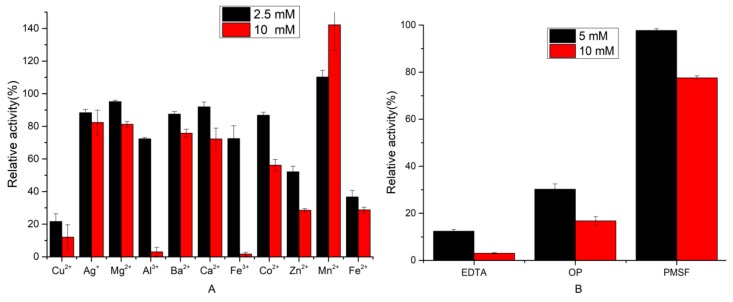
Effects of different metal ions (**A**) and different inhibitors (**B**) on protease activity. Values are expressed as the mean ± SD (*n* = 3).

The effects of inhibitors and metal ions on EH2 activity are shown in Figure 4. The protease disclosed considerable stability (>80%) in the presence of Ca^2+^, Co^2+^, Mg^2+^, Ag^+^, and Ba^2+^. Seawater ions had little effect on protease activity. Mn^2+^ could enhance the activity of EH2 (110–140% relative activity) (Figure 4A). Mn^2+^ plays a vital role in stabilizing enzymes to prevent thermal degradation [15]. However, the activity of EH2 was strongly inhibited by low concentrations of Cu^2+^, Fe^2+^, and Zn^2+^ and high concentrations of Al^3+^ and Fe^3+^. EH2 was sensitive to the metalloprotease inhibitors ethylene diamine tetra acetic acid (EDTA) and 1,10-phenanthroline (OP) (Figure 4B). This result indicated that EH2 was a metalloprotease. Similarly, protease activity was slightly inhibited by 10 mM phenyl methane sulphonyl fluoride (PMSF), showing the involvement of serine residues in the enzyme reaction center.

#### 2.3.3. Effect of Surfactants, Oxidizing Agents, and Organic Solvents on Protease Activity

Similar to the media for the enzyme reaction, organic solvents exerted important advantages in industrial application of food-related conversions and analyses [16]. For this reason, the ability to tolerate various chemicals is critical for a protease to expand its potential for use in the preparation of useful products. As shown in Table 2, reduced protease activity was observed in the presence of SDS (69.57%) and H_2_O_2_ (72.87%). Nevertheless, substantial activity remained (>90%) in the presence of Tween 80 and Triton X-100. Different organic solvents exhibited different effects on protease activity (Table 2). Isoamylol and isopropanol can reduce the activity of EH2. In addition, protease activity was obviously increased by methanol, ethanol, and DMSO (dimethyl sulfoxide). This capability may enhance EH2 as a promising candidate in water-organic solvent systems. 

#### 2.3.4. Hydrolysis of Bovine Serum Albumin and Insulin Box

**Figure 5:**
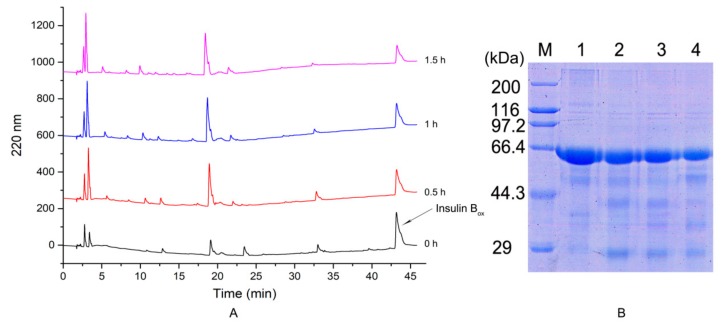
Hydrolysis of bovine serum albumin (BSA) and insulin B_ox_ by EH2. (**A**). Analysis of the hydrolysate of insulin Box by reversed-phase high-performance liquid chromatography. (**B**). Analysis of the hydrolysate of BSA by SDS-PAGE. Line marked 1—20 μL of BSA with 1 μL of EH2, as a control group; line marked 2—20 μL of BSA was incubated with 1 μL of EH2 for 10 min at 50 °C; line marked 3—20 μL of BSA was incubated with 1 μL for enzyme solution for 20 min at 50 °C; line marked 4—20 μL of BSA was incubated with 1 μL of enzyme solution for 40 min at 50 °C.

When the ratio of [E]/[S] was 1:163 in the enzymatic hydrolysis system for 0.5 h, nine product peaks were obtained. With increasing enzymatic hydrolysis time, the peak of insulin Box decreased gradually (Figure 5A). However, the number of product peaks did not increase substantially. It was possible that the abundance of some peaks was reduced because of the hydrolysis of larger peptides into smaller peptides. Proteases with more enzymatic cleavage sites would hydrolyze substrates more effectively, which has contributed to the expansion of their application fields.

After incubation for 20 min or 40 min, the protease could also hydrolyze bovine serum albumin (BSA) (containing 583 amino acids) into a plurality of peptides with varying lengths (Figure 5B). In summary, EH2 with more enzymatic cleavage sites had a good hydrolysis effect on proteins with larger and smaller molecular weights and exhibited high catalytic efficiency.

### 2.4. Mass Spectrum Identification of the Protease

The mass spectrometry method was used for the identification of purified EH2. The results are shown in Table 3. EH2 showed high sequence identity with the metal protease from *Pseudoalteromonas* sp. A28 [17]. The metal protease result was consistent with the conclusion obtained from the inhibitor experiments. 

### 2.5. Preparation of Native Collagen Hydrolytic Peptides

**Figure 6:**
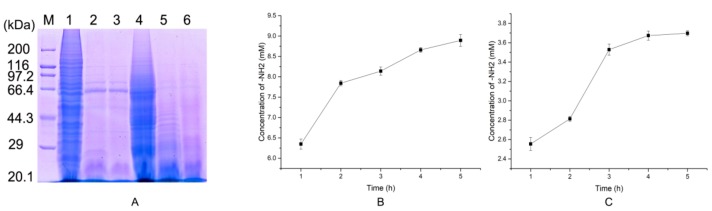
Hydrolysis of native collagen hydrolytic peptides. (**A**). The hydrolysis results after 20 min were analyzed by SDS-PAGE; line marked 1—20 μL of porcine skin collagen (0.3 mg/mL) with 1 μL of EH2; line marked 2—20 μL of porcine skin collagen (0.3 mg/mL) with 1 μL of EH2 for 10 min at 50 °C; line marked 3—20 μL of porcine skin collagen (0.3 mg/mL) with 1 μL of EH2 for 20 min at 50 °C; line marked 4—20 μL of salmon skin collagen (0.4 mg/mL) with 1 μL of EH2; line marked 5—20 μL of salmon skin collagen (0.4 mg/mL) with 1 μL of EH2 for 10 min at 50 °C; line marked 6—20 μL of salmon skin collagen (0.4 mg/mL) with 1 μL of EH2 for 20 min at 50 °C. (**B**). Hydrolysis degree of salmon skin collagen and (**C**) porcine skin collagen treated for 1, 2, 3, 4, and 5 h. Values are expressed as the mean ± SD (*n* = 3).

Collagen structure is defined by three almost identical polypeptide chains. Each polypeptide chain consists of repeating triplets (Gly-X-Y)_n_ [18]. In this study, native collagen from porcine skins and salmon skins was hydrolyzed with EH2. The hydrolysis results after 10 min were analyzed by SDS-PAGE (Figure 6A), and the rate of hydrolysis was also measured (Figure 6B,C). Compared with porcine skin collagen, salmon skin collagen could be hydrolyzed to smaller pieces more effectively by EH2 at low enzyme concentrations. This result showed that the protease could be more suitable for the digestion of marine collagen. The higher the content of Hyp, the stronger the ability for collagen to maintain the three-strand helical structure. The content of Hyp in porcine collagen is significantly higher than that of fish. Thus, porcine skin collagen is harder to hydrolyze. Of course, a large abundance of enzyme active sites may also be one of the reasons for the salmon skin collagen being more adequately hydrolyzed. After 4 h of treatment, the hydrolysis of native collagen did not increase significantly. This result indicated that the hydrolysis had reached a maximum. It is essential for industrial enzymes to possess high enzymatic activity and substrate binding capability, which could shorten the reaction time and release antioxidant peptides more easily. In this respect, EH2 meets our expectations.

### 2.6. Antioxidant Activity of the Hydrolytic Peptides

**Figure 7:**
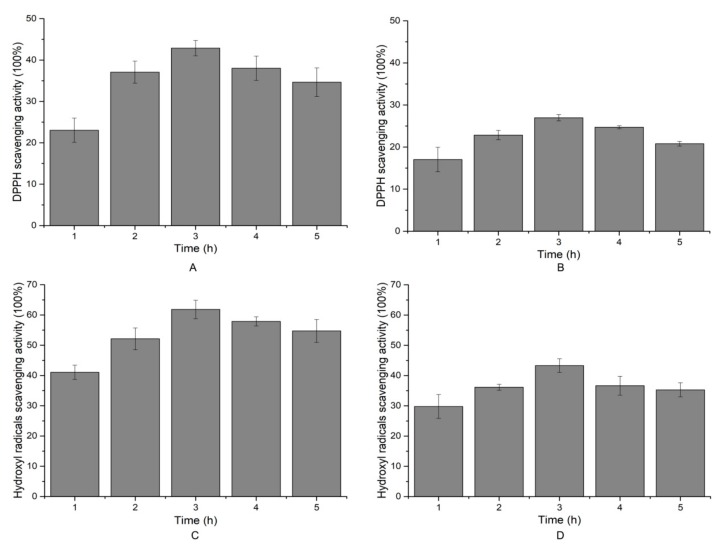
DPPH (1,1-diphenyl-2-picrylhydrazyl) and hydroxyl radical scavenging activity of hydrolysates. (**A**) DPPH and (**C**) hydroxyl radical scavenging activity of salmon skin collagen hydrolysates. (**B**) DPPH and (**D**) hydroxyl radical scavenging activity of porcine skin collagen hydrolysates. Values are expressed as the mean ± SD (*n* = 3).

The performance of antioxidants often varies against different free radicals. DPPH (1,1-diphenyl-2-picrylhydrazyl) radical scavenging, hydroxyl radical scavenging, and ORAC assays (oxygen radical absorbance capacity) were used to assess the antioxidant activities of each hydrolysate fraction every hour. As shown in Figure 7, the DPPH scavenging activity of the two hydrolytic products increased gradually with the time of hydrolysis until the 3 h time point, after which it began to decline. For salmon skin collagen hydrolysates, the strongest DPPH scavenging activity was 42.88% ± 1.85 (Figure 7A). The porcine skin collagen, by contrast, displayed persistent low DPPH scavenging activity during hydrolysis (Figure 7B). DPPH radical scavenging activity is generally used to determine the hydrogen-donating ability of protein hydrolysates [19]. When DPPH encountered a hydrogen-donating substance, the radical was scavenged, and the absorbance at 517 nm was reduced. Our results suggested that salmon skin collagen hydrolysates contained more electron donors (including amino acids or peptides), which could react with free radicals, convert them to more stable products, and terminate the radical chain reaction, when compared with porcine skin collagen hydrolysates.

The highest hydroxyl radical scavenging abilities of 61.83% ± 3.05 and 43.29% ± 2.25 were observed in the salmon skin collagen hydrolysates and porcine skin collagen hydrolysates collected at the 3 h time point, respectively (Figure 7C,D). The hydroxyl radical scavenging ability is a result of the comprehensive action of the reducing power, donation of hydrogen, and the scavenging of active oxygen [20]. The results suggest that two hydrolysates may provide good hydroxyl radical scavenging ability and help to offer a defensive shield against the hydroxyl radical [21].

**Figure 8:**
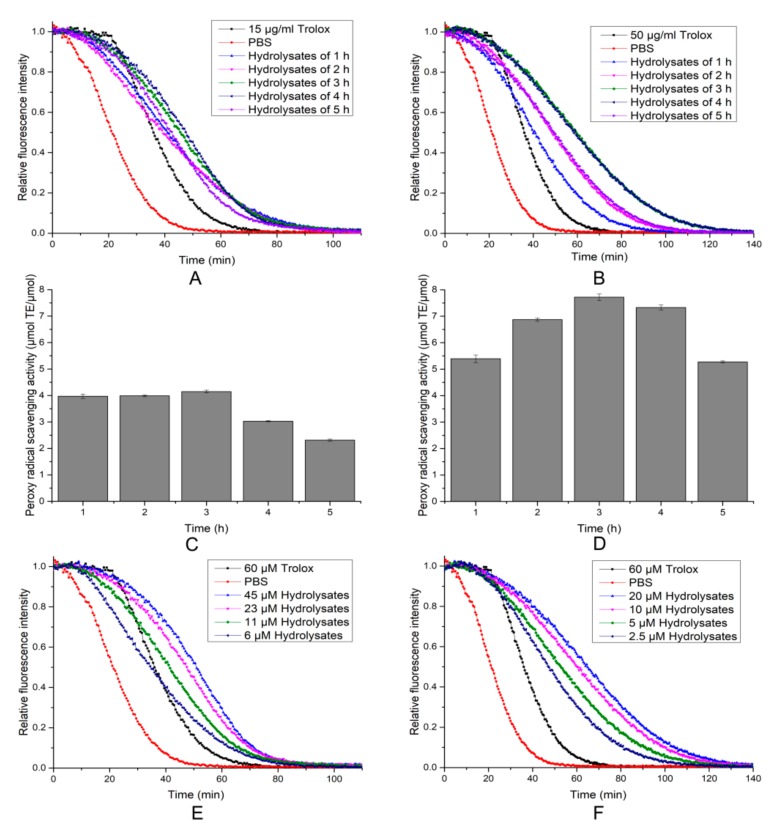
Peroxyl radical scavenging activity (oxygen radical absorbance capacity (ORAC) assay) of hydrolysates. (**A**,**C**) Peroxyl radical scavenging activity of salmon skin collagen hydrolysates with different times. (**B**,**D**) Peroxyl radical scavenging activity of porcine skin collagen hydrolysates at different times. (**E**) Peroxyl radical scavenging activity of salmon skin collagen hydrolysates at different concentrations. (**F**) Peroxyl radical scavenging activity of porcine skin collagen hydrolysates at different concentrations. Values are expressed as the mean ± SD (*n* = 3).

In the ORAC assay, the area under the fluorescence decay curve reflected the quantity of the peroxyl radical removed by peptides. As shown in Figure 8, two hydrolytic products displayed an effect in decreasing the decay of fluorescence, and the antioxidant activity of the porcine skin collagen hydrolysates (7.72 ± 0.13 μmol·TE/μmol) was much stronger than that of the salmon skin collagen hydrolysates (4.15 ± 0.05 μmol·TE/μmol). The results indicated that the porcine skin collagen hydrolysates might contain more active peptides working as hydrogen atom donors [22]. Meanwhile, hydrolysates showed a dose-dependent increase in the inhibition of fluorescence decay (Figure 8E,F).

Based on the DPPH radicals, hydroxyl radicals, and ORAC assays, the two hydrolysis products exerted the best effects when hydrolyzed for 3 h. After 3 h, the antioxidant activity began to decline. The reason may have been that some of the antioxidant peptides were further hydrolyzed with increasing reaction time, given that low molecular peptides contribute more to the inhibitory activity than polypeptides [12,23]. In addition, DPPH and hydroxyl radical scavenging activity of salmon skin collagen hydrolysates were better than those of porcine skin collagen hydrolysates, but as for the absorption capability of against peroxyl radical, the latter had higher efficiency. The results indicated that hydrolysis time and type of substrate exerted important effects on the antioxidant properties of hydrolysates. The ORAC of hydrolysates was not significantly correlated with the DPPH scavenging activity. Therefore, it is necessary to use more than one assay to measure the antioxidant capacity of protein hydrolysates. The antioxidant properties of peptides are greatly influenced by the amino acid composition [24], length [25], and hydrophobicity of the peptide [26]. Therefore, we conclude that the radical scavenging capacities of the hydrolysates might be due to the size and hydrophobic amino acid content of the peptides and the concentration of electron-donating substances in the hydrolysates. Therefore, prepared hydrolysates with appropriate hydrolysis time and source could maximize their functions as radical scavengers.

### 2.7. Cytotoxicity and Intracellular Antioxidant Activity of the Hydrolytic Peptides of the Hydrolytic Peptides on Human Umbilical Vein Endothelial Cells (HUVECs)

**Figure 9:**
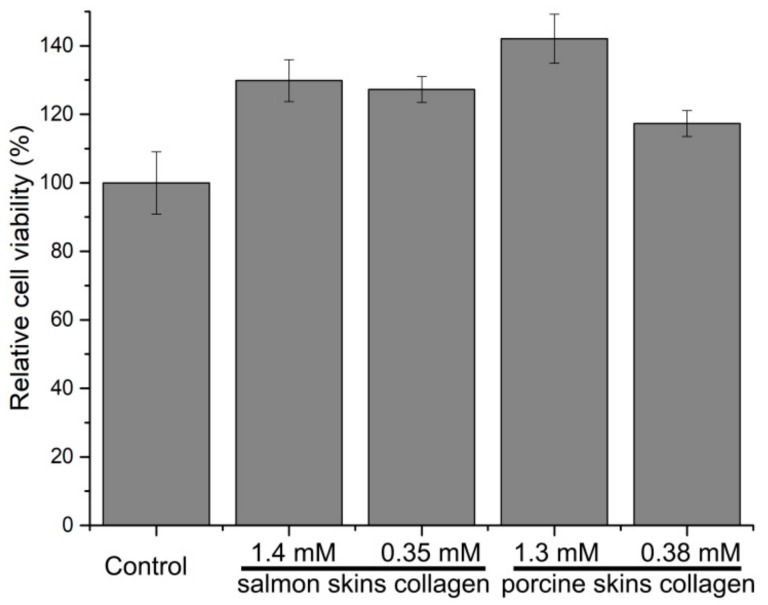
Cell viability as determined by the methylthiazolyldiphenyl-tetrazolium bromide (MTT) assay. Values are expressed as the mean ± SD (*n* = 3).

Endothelial oxidative injury is a key event in the development of many diseases, such as diabetes and arteriosclerosis [27]. Resistance to oxidative damage of endothelial cells will be important in the prevention and treatment of various vascular dysfunction-related diseases. To evaluate the effects of the hydrolytic peptides of two collagens on HUVECs, the methylthiazolyldiphenyl-tetrazolium bromide (MTT) method was used. The results showed that the hydrolytic peptides had no toxicity to cells at 0.35–1.4 mM (Figure 9). In addition, it showed even higher cell viability in the test groups. This confirmed that the hydrolytic peptides were safe and suitable for cell growth and can be used in ROS (reactive oxygen species) experiments.

**Figure 10:**
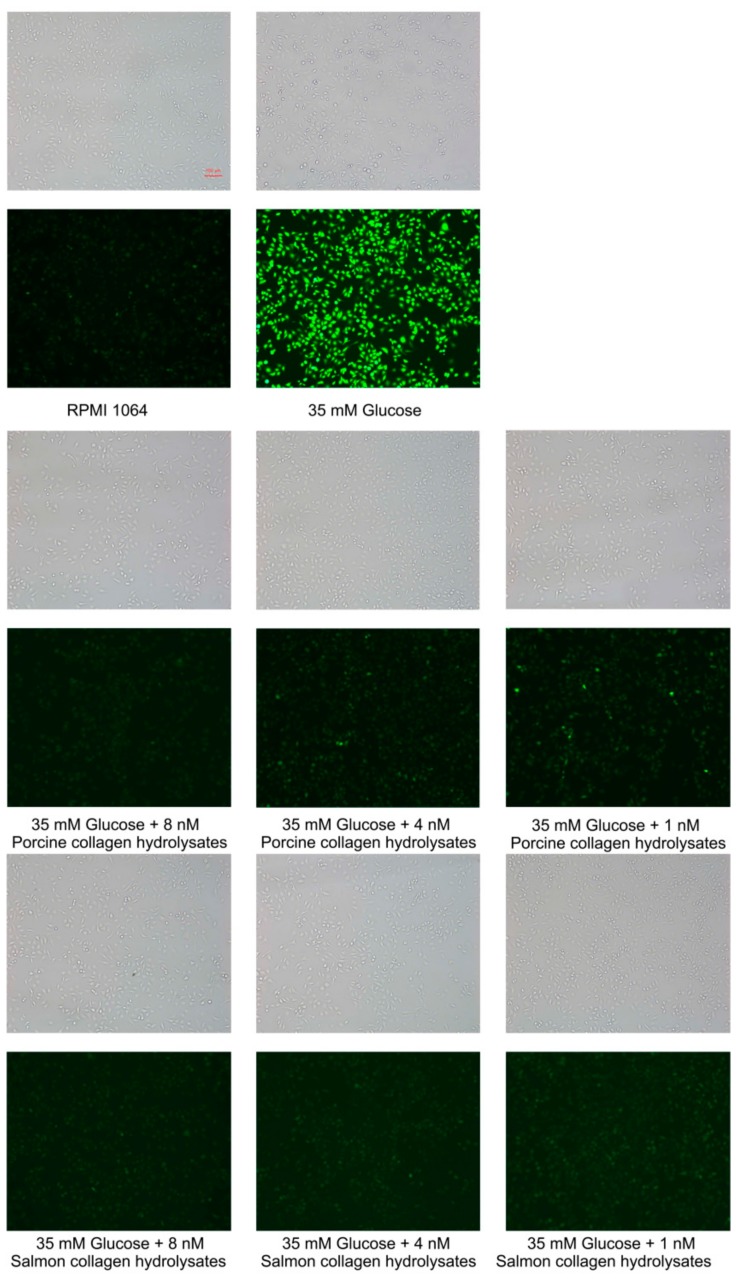
Intracellular reactive oxygen species (ROS) in human umbilical vein endothelial cells (HUVECs) are indicated as green fluorescence by 2,7-dichlorofluorescin diacetate (DCFH-DA).

High glucose could increase oxidative stress in peripheral tissues [28], and increased ROS generation was responsible for the stimulation by high glucose [29]. To investigate the intracellular ROS scavenging effects of the hydrolytic peptides, HUVECs were labeled with DCFH-DA (2,7-dichlorofluorescin diacetate). As shown in Figure 10, cells treated with 35 mM glucose displayed a stronger DCF (2,7-dichlorodihydrofluorescein)-fluorescence intensity than cells in blank groups, which indicated that high glucose could increase oxidative stress in HUVECs. Cells treated with different concentrations of unpurified hydrolytic peptides also displayed low fluorescence intensity. Compared with the high-glucose group, even 1 nM mixed peptides can effectively reduce the levels of intracellular ROS. These results suggested that unpurified hydrolytic peptides attenuated oxidative injury in HUVECs. In addition, the intracellular radical-scavenging effects of 1 nM salmon skin collagen hydrolysates were better than those of porcine skin collagen hydrolysates. At present, research on antioxidant peptides has mainly focused on purified peptides [30]. In fact, as a highly effective food additive, unpurified antioxidant peptides may be preferred because of the high cost and tedious operation of purification.

## 3. Materials and Methods

### 3.1. Materials

Fresh salmon skins and porcine skins were purchased from the seafood market and stored at −20 °C prior to use. The soybean meal, corn powder, and wheat bran were purchased from a supermarket. Tryptone and yeast extract were purchased from Oxoid (Basingstoke, U.K.). Sephadex 75 was purchased from GE Healthcare Life Sciences (Uppsala, Sweden). The other analytically pure regents used are commercially available.

### 3.2. Bacterial Culture

The marine bacterial strains were isolated from the inshore environment of the Bohai Sea and screened with a medium comprising 0.5% tryptone, 0.1% yeast extract, 0.5% casein, 1.5% agar powder, and artificial seawater (pH 7.8). The plates were incubated at 18 °C for 24 h. The formation of a clear hydrolytic zone around the colonies was evaluated as proteolytic activity. All strains were maintained in 2216E agar medium and stored with 20% glycerol at −80 °C.

### 3.3. Preparation of Proteases

The strains were cultured at 18 °C with shaking at 200 rpm in 500 mL flasks with 50 mL of fermentation broth [31]. The culture supernatant was collected by centrifugation (12,000× *g*, 4 °C, 30 min) after 84 h of incubation and stored at −20 °C for the following experiment.

### 3.4. Protease Assay

The protease activity of the culture supernatant to casein was detected by Folin phenol method [1]. In this, 40 μL the purified protease, which was diluted appropriately with 20 mM Tris-HCl (pH 8.0), was mixed with 40 μL 20 mM Tris-HCl (pH 8.0) containing 2% casein (*m*/*v*), and incubated for 10 min at 50 °C. The reaction was stopped by addition of 80 μL trichloroacetic acid (0.4 M), and then centrifuged at 12,000× *g* for 5 min. Then, 30 μL supernatant solution was mixed with 150 μL Na_2_CO_3_ (0.4 M) and 30 μL folin phenol. The mixture was evenly mixed, and stood at room temperature for 10 min, with detected absorbance at 660 nm. One unit of enzyme activity was determined as the amount of enzyme that catalyzed the formation of 1 µg of tyrosine per min.

### 3.5. Protease Purification

The crude enzyme of *Pseudoalteromonas* sp. H2 was precipitated overnight with 40% (NH_4_)_2_SO_4_ at 4 °C. The precipitate was collected by centrifugation, dissolved in 20 mM Tris-HCl (pH 8), and dialyzed. The samples were loaded onto a 5 mL HiTrap^TM^ DEAE column (GE Healthcare) previously equilibrated with 20 mM Tris-HCl (pH 8) and then eluted with a NaCl gradient (0-1 M) in the same buffer at a flow rate of 1 mL /min. Active fractions were collected, concentrated by ultrafiltration (10 kDa MW cut-off membrane, Millipore), and subjected to gel filtration on a Superdex 75 (10 × 300 column 24 mL) previously equilibrated with 20 mM Tris-HCl (pH 8). The flow rate was 1 mL/min, and the active fractions were pooled for further analysis. The protein concentration was determined with a BCA (bicinchoninic acid) kit.

### 3.6. Zymography and SDS-PAGE Analysis

The substrate immersing zymography method and SDS-PAGE were described previously [1]. The electrophoresis voltage for the 5% stacking gel was 100 V, and the 12% running gel was 150 V.

### 3.7. Characterization of the Protease

#### 3.7.1. Effect of Temperature and pH on Protease Activity and Stability

The optimal pH of the protease was determined by different buffers with pH values ranging from 4 to 11 at 50 °C for 20 min. The buffers system was as follows: 50 mM citrate buffer (pH 4.0 to 7.0), 50 mM Tris-HCl buffer (pH 7.0 to 9.0), and 50 mM Gly-NaOH buffer (pH 9.0 to 11.0). The effect of pH on protease stability was determined by measuring the residual activity by the Folin phenol method after the enzyme was incubated with different pH buffers at 4 °C for 24 h. The optimal temperature for the protease was determined by incubating the reaction mixtures in 20 mM Tris-HCl (pH 8) at different temperatures ranging from 20 to 80 °C for 10 min. The thermal stability was determined by measuring the residual activity in 20 mM Tris-HCl (pH 8) after the protease was treated at 50, 60, and 70 °C for 10, 20, 30, 40, 50, and 60 min.

#### 3.7.2. Effect of Inhibitors and Metal Ions on Protease Activity

The effect of metal ions was investigated by measuring the residual activity of the protease after pre-incubation with 2.5 and 10 mM Ca^2+^, Co^2+^, Cu^2+^, Mg^2+^, Mn^2+^, Zn^2+^, Ag^+^, Al^3+^, Ba^2+^, Fe^3+^ and Fe^2+^ for 30 min. In addition, 5 and 10 mM phenyl methane sulphonyl fluoride (PMSF), ethylene diamine tetra acetic acid (EDTA), and 1,10-phenanthroline (OP) were used to assess the effect of protease inhibitors. All assays were carried out in 20 mM Tris-HCl (pH 8) and at 50 °C. The activity of samples without metal ions and inhibitors was set at 100% activity as a control.

#### 3.7.3. Effect of Surfactants, Oxidizing Agents, and Organic Solvents on Protease Activity

The effects of surfactants (SDS, Tween 80, and Triton X-100) and the oxidizing agent H_2_O_2_ were also analyzed. The protease was preincubated with different agents for 1 h at room temperature. The compatibility of the protease with organic solvents was evaluated with acetone, dimethyl sulfoxide (DMSO), ethanol, isopropanol, methanol, and isoamyl alcohol (25% (*v*/*v*)). The protease was preincubated with organic solvents for 5 h at room temperature, and the protease residual activity was measured. Residual protease activity was determined in 20 mM Tris–HCl (pH 8) at 50 °C.

### 3.8. Hydrolysis of Bovine Serum Albumin and Insulin Chain B by the Protease

The stock solution of insulin Box (10 mg/mL) was prepared by dissolving 100 µg of insulin Box (Sigma-Aldrich) in 10 µL of 0.01 M HCl. Insulin Box substrate (2 mg/mL) and enzyme solution (0.1 mg/mL) were mixed and incubated in 20 mM Tris-HCl (pH 8) for a certain time at 50 °C, and then 1 μL of 10% trifluoroacetic acid (TFA) was added to terminate the reaction. The samples were loaded onto a C18 reversed-phase column previously equilibrated with 2% methanol (containing 0.1% TFA) and then eluted with a methanol gradient (2–100%) at a flow rate of 0.5 mL/ min. The detection wavelength was 220 nm.

Twenty microliters of bovine serum albumin solution (BSA, 1 mg/mL) with 1 μL of enzyme solution (0.2 mg/mL) was mixed and incubated for 10 min, 20 min, and 40 min at 50 °C, and electrophoresis was performed after thermal denaturation.

### 3.9. Mass Spectrum Identification of the Protease

The protease was identified by mass spectrometry. These proteins were excised from the polyacrylamide gel and digested in situ with trypsin in digestion buffer (ammonium bicarbonate 100 mM, pH8.5). The peptides from the digestion were extracted out with acetonitrile, and completely dried down in a SpeedVac device (Thermo Fisher, Waltham, Massachusetts, USA). Then, the dried sample was redissolved in sample solution (2% acetonitrile, 97.5% water, 0.5% formic acid). The protein solution was reduced by DTT (dithiothreitol) and all cysteine residues alkylated by iodoacetamide and cleaned. After this, a dissolved peptide sample was analyzed by a NanoLC-ESI-MS/MS system (Nanoscale reversed-phase liquid chromatography-electrospray ionization-tandem mass spectrometry). The mass spectrometric data was used to search against the most recent non redundant protein database (Non-Redundant database, National Center for Biotechnology Information (NCBI)) with ProtTech’s ProtQuest software suite. Detailed experimental steps have been described previously in another paper by the authors [32].

### 3.10. Preparation of Collagen Hydrolytic Peptides

Native collagen was extracted from porcine skins and salmon skins according to the method of Wu [33]. Native collagen (0.25 g) was weighed and dissolved in 50 mL of 10 mM PBS (phosphate buffer saline). The protease (100 μL, 0.2 mg/mL) was mixed and incubated with native collagen (2 mL, 5 mg/mL) at 50 °C for 1 h. Collagen hydrolytic peptides were collected. The free amino acid content was estimated by the indene tri-ketone colorimetric method [31,33].

### 3.11. DPPH Radical Scavenging, Hydroxyl Radicals Scavenging and ORAC Activity Assay

The DPPH radical scavenging, hydroxyl radical scavenging, and ORAC activity assays were measured according to the method of Wu [31]. A mixed solution of hydrolytic peptides and DPPH (the ratio of hydrolysates to DPPH was 1:5) was sealed and incubated for 60 min at 37 °C in the dark, and the decrease in the absorbance at 517 nm was measured against ethanol with an Enspire spectrophotometer (Perkin Elmer, Waltham, MA, USA).

In the hydroxyl radical scavenging activity assay, an FeSO_4_ solution (40 μL, 2 mM), 1,10-phenanthroline (OP, 40 μL, 2 mM), and the sample (80 μL) were mixed. Then, H_2_O_2_ (40 μL, 0.03% *v*/*v*) was added to initiate the reaction. The absorbance of the mixed solution was measured at 536 nm after incubation at 37 °C for 60 min. The group without any antioxidant was used as the negative control, and the mixture without H_2_O_2_ was used as the blank.

In the ORAC activity assay, sample solution (20 μL), and fluorescein (150 μL, 96 nM) were added to a 96-well plate and preincubated at 37 °C in the Enspire spectrophotometer. Then, AAPH (2,2′-azobis-2-methyl-propanimidamide, dihydrochloride) (30 μL, 320 mM) was added to initiate the reaction. The reaction was performed at 37 °C. The fluorescence intensity was measured every 60 s for 150 cycles with excitation and emission wavelengths of 485 nm and 538 nm, respectively. Vitamin E was used as a positive control.

### 3.12. The Cytotoxicity of Collagen Hydrolytic Peptides by MTT Methods

Human umbilical vein endothelial cells (HUVECs) (1 × 10^5^ cells/mL) were plated in 96-well plates and cultured in RPMI (Roswell Park Memorial Institute) -1640 medium with 10% (*v*/*v*) fetal bovine serum (FBS) at 37 °C in a humidified atmosphere of 5% CO_2_ for 12 h and then treated with different concentrations of hydrolytic peptides (0.35-1.4 mM) for 12 h. The concentration of hydrolytic peptides was diluted with 10 mM PBS. The effects of collagen hydrolytic peptides on the growth of cells were evaluated by a methylthiazolyldiphenyl-tetrazolium bromide (MTT) assay [30].

### 3.13. Determination of ROS Level

The level of intracellular ROS (reactive oxygen species) was determined by DCFH-DA (2,7-dichlorofluorescin diacetate). HUVECs (1 × 10^5^ cells/mL) were cultured in 24-well plates for 12 h. Then, the medium was replaced by RPMI 1640 medium (without FBS, but with 35 mM glucose and different concentrations of peptides) and incubated for 12 h. Subsequently, RPMI 1640 medium with 1/10^3^ DCFH-DA (*v*/*v*) was added to each well and incubated for 1 h in a cell incubator. Excess DCFH-DA was washed away with 10 mM PBS, and images of stimulated HUVECs were collected using a Nikon ECLIPSE TE2000-U with a digital CCD (charge couple device) camera (DS-U2, Nikon, Japan) under fluorescence.

### 3.14. Statistical Analysis

All experiments were repeated three times (*n* = 3). The values were expressed as the mean ± standard deviation, which were calculated with Origin 9.1 software (Northampton, Massachusetts, USA). An ANOVA test was used to analyze data in SPSS 19.0 software.

## 4. Conclusions

The study presented a report on the isolation of a metalloprotease from *Pseudoalteromonas* sp. H2. Proteases with high tolerance to pH, temperature, and organic solvents were effective in the preparation of antioxidant peptides by hydrolysis of porcine and salmon skin collagen. Moreover, our results showed that DPPH and hydroxyl radical scavenging activity of salmon skin collagen hydrolysates were better than those of porcine skin collagen hydrolysates. In addition, for the intracellular radical-scavenging effects of the low concentration of hydrolysis, the former were also superior to the latter. However, as for the absorption capability of the peroxyl radical, the latter had higher efficiency. Hydrolysis time and type of substrate exerted important effects on the antioxidant properties of hydrolysates. The performance of antioxidants often varies for different free radicals. The ORAC of hydrolysates was not significantly correlated with the DPPH scavenging activity. In summary, the protease EH2 has multiple tolerance and efficient hydrolysis capacity, indicating that it could be a potential application in the biocatalysis industry. In addition, the hydrolyzed peptides from collagen prepared by H2 have good antioxidant activity, indicating that it could be a potential additive in the food processing industry and cosmetics industry. Of course, to provide indications of potential health benefits, in vivo studies are required and are on the way.

## Figures and Tables

**Table 1:** Protease activity in purification process of EH2.

Purification Stage	Total Protein	Total Enzyme Activity	Specific Activity
	(mg)	(U)	(U/mg)
Crude enzyme	350	3678	10.5
Ammonium sulfate precipitation	50.2	2326	46.3
Anion exchange	5.4	1597	295.7
Size exclusion	2.1	998	475.2

**Table 2:** Activity and stability of EH2 in the presence of surfactants, oxidizing agents, and organic solvents.

Detergents	Residual Activity (%)
none	100
SDS (0.5%)	69.57 ± 1.55
Triton X-100 (1%)	91.86 ± 4.84
Tween 80 (1%)	104.94 ± 1.84
H2O2 (1%)	72.87 ± 1.55
Acetone (25% (*v*/*v*))	93.22 ± 5.72
dimethyl sulfoxide (DMSO) (25% (*v*/*v*))	128.08 ± 2.73
ethanol (25% (*v*/*v*))	110.21 ± 3.35
isopropanol (25% (*v*/*v*))	60.92 ± 2.46
methanol (25% (*v*/*v*))	115.49 ± 0.70
isoamyl alcohol (25% (*v*/*v*))	78.96 ± 2.73

**Table 3:** Amino acid sequence of the protease by mass spectrum.

Peptide Mass	Peptide Sequence	Sequence Header	Similarity (%)	Mr (calc)
1491	^386^GSNDWLVGQEIFK^399^	metal protease [*Pseudoalteromonas* sp. A28]	100	35 kDa
1711	^439^AFYNLATTPGWDTQK^453^	metal protease [*Pseudoalteromonas* sp. A28]	100	
2093	^386^GSNDWLVGQEIFKGNGALR^405^	metal protease [*Pseudoalteromonas* sp. A28]	100	
2240	^366^SGGLNEAFSDMAGEAAEFYMK^386^	metal protease [*Pseudoalteromonas* sp. A28]	100	
